# Two Cases of Retroperitoneal Liposarcoma Diagnosed Using Endoscopic Ultrasound-Guided Fine-Needle Aspiration (EUS-FNA)

**DOI:** 10.1155/2009/673194

**Published:** 2009-10-22

**Authors:** Yuta Takahashi, Atsushi Irisawa, Manoop S. Bhutani, Takuto Hikichi, Tadayuki Takagi, Goro Shibukawa, Takeru Wakatsuki, Hidemichi Imamura, Ai Sato, Masaki Sato, Tsunehiko Ikeda, Rei Suzuki, Katsutoshi Obara, Yuko Hashimoto, Kazuo Watanabe, Hiromasa Ohira

**Affiliations:** ^1^Department of Gastroenterology and Rheumatology, Fukushima Medical University School of Medicine, 1 Hikarigaoka, Fukushima 960-1295, Japan; ^2^Department of Gastroenterology, Hepatology and Nutrition, UT MD Anderson Cancer Center, Houston, TX 77030-4009, USA; ^3^Department of Endoscopy, Fukushima Medical University Hospital, 1 Hikarigaoka, Fukushima 960-1295, Japan; ^4^Department of Pathology, Fukushima Medical University Hospital, 1 Hikarigaoka, Fukushima 960-1295, Japan

## Abstract

This report describes our experience with two cases that were ultimately diagnosed as retroperitoneal liposarcoma using endoscopic ultrasound-guided fine-needle aspiration (EUS-FNA). Case 1 is that of a 54-year-old woman with chief complaints of nausea and abdominal distention. Computed tomography (CT) and magnetic resonance imaging (MRI) revealed a large (15 cm diameter) tumor, which was significantly compressing the stomach and apparently occupied the entire left abdominal cavity. Although advanced primary gastrointestinal stromal tumor (GIST) or retroperitoneal tumor was inferred as the differential diagnosis, a definitive diagnosis was difficult using imaging alone. After EUS-FNA was done, the tumor was diagnosed histopathologically as high-grade liposarcoma. Case 2 is that of a 73-year-old man. Abdominal ultrasonography and CT showed a 6 cm diameter tumor within the pelvic cavity. The tumor had high MRI signal-intensity on both T1 and T2 images. Endorectal EUS showed a hyperechoic mass. The images suggested lipoma or liposarcoma containing lipoma-like components. Myxoid liposarcoma was revealed by subsequent EUS-FNA. Performing EUS-FNA was clinically useful for determining the subsequent therapeutic strategy in these cases where a tumor of unknown origin existed in the retroperitoneum.

## 1. Introduction

Endoscopic ultrasonography (EUS) is an important modality in the evaluati of luminal gastrointestinal diseases as well as diseases of the pancreas, gallbladder, and biliary ductal system. Endoscopic ultrasound-guided fine-needle aspiration (EUS-FNA) has been developed as a diagnostic tool using EUS [1]. EUS-FNA, which can support an accurate pathological diagnosis, has become an important minimally invasive tissue sampling modality in a variety of clinical situations [2].

 Although liposarcoma is a malignant soft-tissue tumor that frequently occurs in extremities and retroperitoneum [3], it reportedly occurs rarely in the GI tract in locations such as the stomach, pancreas, mesenterium, and greater omentum. Because of the retroperitoneal location of these tumors, it is often difficult to collect pathological samples nonsurgically with the final pathology not uncommonly established only at the time of surgical exploration. Few reports have described the preoperative diagnoses of retroperitoneal liposarcoma using percutaneous FNA [4]. However, no report has described liposarcoma of the retroperitoneum diagnosed by EUS-FNA. We report two cases in which such lesions occurred primarily in the retroperitoneum which were ultimately diagnosed as liposarcoma using EUS-FNA.

## 2. Case Report

**Case 1**

**Figure 1:**
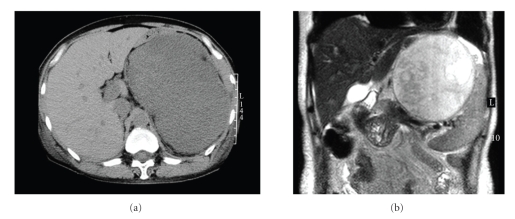
(a) Abdominal CT showing a huge tumor as a low-density mass occupying much of the left side of the abdominal cavity. (b) Sagittal T2-weighted MR image showing a mosaic-patterned high-intensity mass, with an enclosed low-intensity septum.

**Figure 2:**
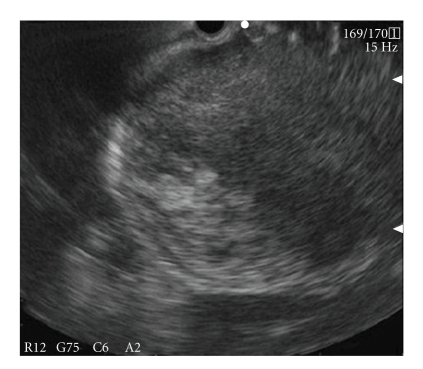
A mass was identified as hypoechoic with some partial hyperechoic lesions suggesting hemorrhage within the tumor on EUS. EUS-FNA was performed using the transgastric approach.

**Figure 3:**
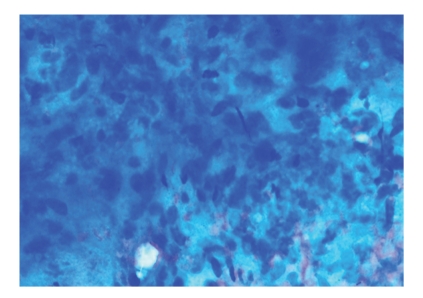
Histopathological examination revealed a few signet-ring-cell-like lipoblasts and several pleomorphic cells manifested in a myxoid background (Giemsa stain, ×60).

A 54-year-old woman who suffered from nausea and abdominal distention had consulted with her family doctor. She had no previous history of a related disease or these symptoms. Esophago-gastro-duodenoscopy (EGD) showed a subepithelial mass on the upper gastric corpus; an abdominal CT showed a huge tumor as a low-density mass occupying most of the left side of the abdominal cavity (Figure 1(a)). She was referred to our department for further evaluation of the abdominal tumor. Blood analysis results revealed Hemoglobin: 8.3 g/dL (13.2–16.8). Both CEA and CA19-9 were within their normal range. Abdominal ultrasonography showed a 15 cm diameter tumor on the left side of the abdominal cavity from the abdominal centromedian. Even though the internal part was almost hypoechoic, dotted hyperechoic spots were also apparent, suggesting possible intratumoral hemorrhage. Abdominal MRI displayed the tumor as a high-intensity image on the T1 and as a mosaic-patterned high-intensity image on T2 images, showing a septum within it (Figure 1(b)). Results of EUS showed a hypoechoic area with some partial hyperechoic lesions, suggesting hemorrhage within the tumor (Figure 2). Although these findings suggested the possibility of a gastrointestinal stromal tumor (GIST) or retroperitoneal tumor, a definitive diagnosis based only on those images was considered impossible. For that reason, EUS-FNA was done using the transrectal approach with a 19-gauge trucut needle (Quick-Core; Wilson-Cook Medical Inc.) and 19-gauge aspiration needles (Echo-Tip; Wilson-Cook) with a cytopathologist in attendance (the role of onsite pathologist is to assess whether adequate materials for histocytopathological diagnosis were obtained or not). At first, the trucut needle was used twice. However, the adequate materials were able to obtain because of the tumor condition (necrosis and bleeding). Thus, 19G aspiration needle was used in what follows. Four times needling was done, and then the adequate samples for cytology were obtained. Cytopathological examination of the sample obtained by EUS-FNA revealed spindle cells as well as myxoid and round cells (Figure 3). Additional immunostaining showed that the c-kit and desmin were negative. High-grade liposarcoma was diagnosed from the specimens obtained by EUS-FNA. No aggressive treatment was done according to the patient's opinion.

**Case 2**

**Figure 4:**
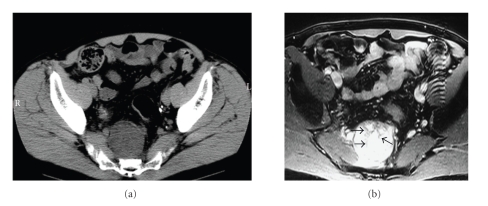
(a) CT showing a 6 cm diameter low-density tumor within the pelvis. (b) Axial T2-weighted MR image showing a very-high-intensity mass with a low-intensity septum (arrows).

**Figure 5:**
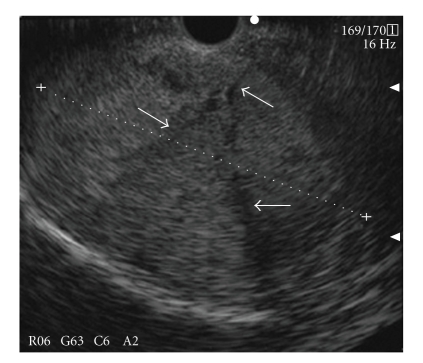
EUS showed that the internal echo was homogeneously hyperechoic and the hypoechoic septum (arrows) was also apparent. Subsequently EUS-FNA was performed using a transrectal approach.

**Figure 6:**
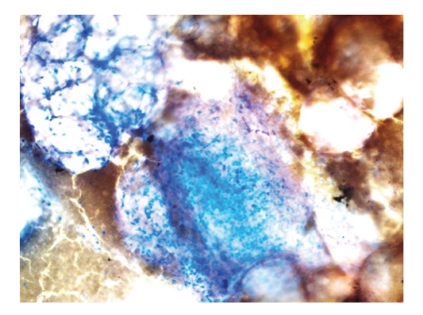
Histopathological examination revealed lipoblasts of various sizes and spindle cells with slightly atypical nucleus manifested against a myxoid background (Giemsa stain, ×60).

A 73-year-old man who showed a retroperitoneal mass on abdominal ultrasonography for health screening visited our hospital for further examination. Results of CT showed a 6 cm diameter low-density tumor within the pelvis (Figure 4(a)). Abdominal MRI showed a tumor detected as high-intensity on both T1 and T2 images with a low-intensity septum (Figure 4(b)). Subsequent EUS showed that the internal echo was homogeneously hyperechoic and the hypoechoic septum was also apparent (Figure 5). These findings suggested that the tumor could be a lipoma or liposarcoma. For further management, EUS-FNA was performed using a transrectal approach with four passes (19 gauge trucut/aspiration needles: Quick-Core and Echo-Tip). As in Case 1, adequate materials were not obtained using trucut needle because of necrosis in the tumor. Therefore, 19G/22G aspiration needle was used. Since the adequate materials were not taken using 19G needle due to tumor condition (necrosis and bleeding), 22G needle was used. Four times needlings using 22G needle were done, and then the adequate samples were taken. Histopathological examination using EUS-FNA samples revealed many lipoblasts and spindle cells accompanying cytonuclear atypia. In addition, some spindle cell clusters manifested a myxoid background (Figure 6). These findings supported the diagnosis of this case as a myxoid liposarcoma. No aggressive treatment was done according to the patient's opinion.

## 3. Discussion

Liposarcoma is a kind of sarcoma that preferentially develops in the extremities (40%), retroperitoneal space (19%), and groin (12%), accounting for 9.8–16% of all sarcomas [5–7]; with most patient aged between 40 and 60 [3]. Since these tumors in the earlier stages may be asymptomatic with no significant laboratory abnormalities, such tumors have often grown to a large size by the time they are identified using a diagnostic modality such as US or CT.

Histopathologic classification of liposarcomas was proposed by WHO classification [8] in 1969, based on the classification of Enzinger and Winslow [9], as five groups: well-differentiated type, myxoid type, round-cell type, pleomorphic type, and mixed type. In 1979, Evans [10] reported a dedifferentiated type, a mixture of high-grade sarcoma components in the well-differentiated type of liposarcoma, which is classified as a kind of subspecific well-differentiated type. Now, it has extended its range sufficiently to contain mixtures of other low-grade sarcoma components [11]. The prognosis of liposarcoma is presumed to be related closely to its tissue type. Enzinger and Winslow [9] report that although the respective survival rates of well-differentiated and myxoid types at five years are 85% and 77%, those of round-cell type and pleomorphic types have a significantly lower survival: 21% and 18%, respectively. Consequently, the well-differentiated and the myxoid types due to their good prognosis are classified into the low-grade group. The round-cell type and pleomorphic types due to the poor prognosis are classified into the high-grade group. The survival rate of the undifferentiated type at five years is also poor; some report that it is as low as 30% [11]. Since this type also has poor prognosis, it is, therefore, classified into the high-grade liposarcoma group (Table 1).

As for treatment of liposarcomas, surgical therapy is the first option in resectable cases. Retroperitoneal liposarcoma has few clinical symptoms. Therefore, it often infiltrates into the surrounding organs. Consequently, the local recurrence rate is as high as 60% at five years. Decreasing the risk of recurrence requires en bloc excision including as much surrounding tissue as possible to prevent remnant tumor tissue [12, 13]. For unresectable or advanced cases, or as a supplemental postoperative therapy, chemotherapy combined with adriamycin, cycophosphamide, doxorubicin, vincristine, dacarbazine, methotrexate, or ifosfamide at times has shown promising results. Some reports have shown that poorly differentiated tumors are treated more successfully than well-differentiated ones [14] In addition, radiation therapy is sometimes given as supplemental therapy particularly for differentiated and myxoid types because their sensitivity to radiation is higher than other types [15].

EUS guided FNA has been applied for cytopathological diagnosis of a variety of lesions within and outside the gastrointestinal tract. Although there is a case of pancreatic metastasis of liposarcoma diagnosed by EUS-FNA [16], to our knowledge, no cases have been reported in which retroperitoneal liposarcoma was definitively diagnosed using EUS-FNA. As described earlier, various imaging modalities such as CT and MRI are the initial cross-sectional imaging modalities for imaging liposarcoma and other soft tissue tumors. Although these cross sectional imaging modalities are effective for diagnosis of invasion or metastasis, the imaging findings may overlap among various soft tissue tumors and might not yield a precise diagnosis. The second case presented herein had high-signal-intensity on both T1 and T2 weighted images. Although the tumor was suggested to contain lipoma-like tissues and was suspected to be liposarcoma, preoperative diagnosis in the first case was difficult. In addition, the EUS showed that the mass was heterogeneously hypoechoic to hyperechoic. The differences in imaging characteristics of liposarcomas are often attributed to characteristics of this tumor, which is prone to internal bleeding and necrosis. The well-differentiated type has more lipoma-like tissues, with high-signal intensity on T1 and T2. On the other hand, poorly differentiated types have fewer lipoma-like tissues, with increasing internal bleeding or necrosis making a precise diagnosis on imaging alone a bit difficult. Because this tumor's response to treatment and prognosis varies depending on the level of differentiation, biopsy as a final diagnosis is important. Thus, the usefulness of percutaneous CT/US- and transgastrointestinal EUS-guided FNA for liposarcoma has been reported [4, 16]. Although some reports have suggested that it is difficult to diagnose liposarcoma with FNA [17], the rapid on-site cytological evaluation during the procedure can reduce inadequate samples and engender a correct diagnosis by EUS-FNA [18, 19]. In addition, the choice of methodology for obtaining the material, aspiration biopsy or trucut biopsy, is also important. We believe that using of aspiration biopsy needle is recommended to diagnose the lesion which is suspected liposarcoma on imaging modalities even if the mass is large, but not core biopsy needle, in our experiences.

## Figures and Tables

**Table 1:** Histopathologic classification of liposarcoma.

Grade	Classification	Prognosis (survival rates at 5 years)
Low-grade	Well-differentiated [8, 9]	85%
	Myxoid type [8, 9]	77%

High-grade	Round-cell type [8, 9]	21%
	Pleomorphic type [8, 9]	18%
	Dedifferentiated type [10, 11]	30%
